# Antagonistic interaction between two key endodontic pathogens *Enterococcus faecalis* and *Fusobacterium nucleatum*

**DOI:** 10.1080/20002297.2022.2149448

**Published:** 2022-11-25

**Authors:** Doudou Xiang, Pu-Ting Dong, Lujia Cen, Batbileg Bor, Renate Lux, Wenyuan Shi, Qing Yu, Xuesong He, Tingxi Wu

**Affiliations:** a Department of Operative Dentistry and Endodontics, School of Stomatology, The Fourth Military Medical University, Xi’an, Shaanxi, China; b Department of Microbiology, The Forsyth Institute, Cambridge, Massachusetts, USA; c Department of Oral Medicine, Infection and Immunity, Harvard School of Dental Medicine, Boston, Massachusetts, USA; d School of Dentistry, UCLA, Los Angeles, California, USA

**Keywords:** Interspecies interaction, coaggregation, *Enterococcus faecalis*, *Fusobacterium nucleatum*, *fap2*, H_2_O_2_

## Abstract

**Background:**

Endodontic infections are known to be caused by pathogenic bacteria. Numerous previous studies found that both *Fusobacterium nucleatum* and *Enterococcus faecalis* are associated with endodontic infections, with *Fusobacterium nucleatum* more abundant in primary infection while *Enterococcus faecalis* more abundant in secondary infection. Little is known about the potential interactions between different endodontic pathogens.

**Objective:**

This study aims to investigate the potential interaction between *F. nucleatum* and *E. faecalis* via phenotypical and genetic approaches.

**Methods:**

Physical and physiological interactions of *F. nucleatum* and *E. faecalis* under both planktonic and biofilm conditions were measured with co-aggregation and competition assays. The mechanisms behind these interactions were revealed with genetic screening and biochemical measurements.

**Results:**

*E. faecalis* was found to physically bind to *F. nucleatum* under both *in vitro* planktonic and biofilm conditions, and this interaction requires *F. nucleatum fap2*, a galactose-inhibitable adhesin-encoding gene. Under our experimental conditions, *E. faecalis* exhibits a strong killing ability against *F. nucleatum* by generating an acidic micro-environment and producing hydrogen peroxide (H_2_O_2_). Finally, the binding and killing capacities of *E. faecalis* were found to be necessary to invade and dominate a pre-established *in vitro F. nucleatum* biofilm.

**Conclusions:**

This study reveals multifaceted mechanisms underlying the physical binding and antagonistic interaction between *F. nucleatum* and *E. faecalis*, which could play a potential role in the shift of microbial composition in primary and secondary endodontic infections.

## Introduction

Endodontic infections are categorized as primary and secondary infection according to the time when microbial infection occurs [1]. Primary infection is caused by invasion and colonization of the pulp by oral microbes, while secondary infection typically results from a persistent microbial root canal infection after initial endodontic treatment due to retention or introduction of microbes during treatment of primary infection [2].

Many studies have characterized microbes associated with primary and secondary infections and established that they harbor distinct microbial communities [1–3]. Based on current knowledge, *Fusobacterium nucleatum* is mainly associated with primary endodontic infection, and often detected in high prevalence and high abundance [3–9]. While the prevalence of *F. nucleatum* in secondary infection remains relatively high, its abundance drops markedly [8,10,11]. In contrast, *Enterococcus faecalis* is rarely detected in primary infection, but during secondary infection, its prevalence increases to 20–77% in general while its abundance could be as high as 90% [8,11–15]. These intriguing findings promoted us to investigate the potential interaction between *F. nucleatum* and *E. faecalis* in the current study.

Both *E. faecalis* and *F. nucleatum* have been extensively studied as individual pathogens [15–19]. *E. faecalis* is a Gram-positive facultative anaerobe that naturally inhabits the human gastrointestinal tract [20,21]. Known virulence functions of *E. faecalis* include its ability to penetrate dentin tubules to establish biofilms and to survive for a prolonged period in harsh environments such as low pH, low nutrition, and low oxygen [15,16,22]. *F. nucleatum* is an oral Gram-negative strict anaerobe with stringent requirements for growth and survival [23,24]. *F. nucleatum* plays a key role as a ‘bridging’ organism to promote oral biofilm formation [25]. *F. nucleatum*’s connection with endodontic infection also includes virulence factors, such as dysregulation of inflammasomes in dental pulp cells [26]. Except for an early report on the possible *in vitro* co-aggregation between *F. nucleatum* and *E. faecalis* [27], the interaction between these two important oral pathogens remains largely unexplored. In this study, we used physiological and genetic approaches to characterize the interaction between *E. faecalis* and *F. nucleatum*.

## Methods

### Bacterial strains and culture conditions

*F. nucleatum* wild type strain ATCC 23726 and its mutant derivatives defective in outer membrane autotransporter proteins, including *Fn*1449(*fap2*), *Fn*1526(*radD*), *Fn*2058(*aim1*), *Fn*2047, *Fn*0254, *Fn*1554, *Fn*1253(*aid1*), and *Fn*1893 [28], were maintained under anaerobic conditions (10% H_2_, 10% CO_2_, 80% N_2_) at 37°C on either Columbia agar supplemented with 5% sheep blood or in Columbia Broth (CB; BD Difco, Detroit, MI, USA). Thiamphenicol (MP Biomedicals, Irvine, CA) at 5 μg per mL was used for selection and maintenance of *F. nucleatum* mutant strains possessing the *catP* determinant. *E. faecalis* wild type strain OG1RF (ATCC 47077) and clinical strain DX1 (isolated by Dr. Xuesong He’s laboratory) were grown aerobically at 37°C on brain-heart infusion (BHI; BD Difco, Detroit, MI, USA) agar plates or broth. For two-species cocultures, we used JVN (Josamycin, Vancomycin and Norfloxacin) selective agar plates in anerobic conditions to grow and count *F. nucleatum* cells [29] and used BHI plates in aerobic conditions to grow and count *E. faecalis* cells.

### Coaggregation assay

Coaggregation visual assays were performed according to the following protocol [28]: exponential phase cultures of *E. faecalis* and *F. nucleatum* cells were washed with and re-suspended in Coaggregation buffer (CAB, 150 mM NaCl, 1 mM Tris, 0.1 mM CaCl_2_, 0.1 mM MgCl_2_·H_2_O; pH 7.5) to a final concentration of 2 × 10^9^ cells per mL. Suspensions of strains to be examined for coaggregation were combined with an equal volume of a test strain adjusted to the same cellular concentration in CAB to a total volume of 1 mL in a reaction tube. The reaction mixtures were immediately vortexed for 10s and incubated for 120 min. The evaluation was performed using a visual scoring system ranging from 0 to 4. A score of 0 was assigned for no visible coaggregation and a score of 4 described complete sedimentation of strains with a clear supernatant [30].

For the coaggregation inhibition assay, either D-galactose, D-Mannose, L-arginine, L-leucine, L-glutamic acid was added to the reaction tube containing only *F. nucleatum* cells to a final concentration of 100 mM. The suspension was then vortexed and incubated for 5 min prior to the addition of the coaggregation test partner. Once the partner strain was added, the reaction mixture was vortexed again and the assay was evaluated by the quantitative coaggregation assay. The final concentration of each inhibitor per coaggregation reaction was 50 mM [31].

The quantitative (spectrophotometric) coaggregation assays were performed similar as the visual assay described above with the following additional steps: optical density measured at 600 nm (OD600) of reaction mixtures were obtained spectrophotometrically. After 120 min of incubation, reaction mixtures were centrifuged at low speed (100 g for 1 min) to pellet the co-aggregated cells while leaving non-aggregated bacteria in suspension. OD600 of the supernatants were measured after the 120 min incubation. Relative coaggregation of species A and B was determined by dividing the difference between the total turbidity of each partner strain and the coaggregation supernatant turbidity by the total turbidity of each partner strain using the formula: {[OD600(A)+OD600(B)]−OD600(A + B)}/[OD600(A)+OD600(B)] [32].

### Competition assay

The competition assays on agar plates between *E. faecalis* and *F. nucleatum* were performed using a previously established protocol [33]. For competition assays under planktonic conditions, exponential phase cultures of *E. faecalis* and *F. nucleatum* were resuspended in pre-reduced fresh CB to a final OD600 of 0.1, equivalent to approximately 1 × 10^8^ cells per mL. An equal volume of *E. faecalis* and *F. nucleatum* were mixed, and cultures were incubated at 37°C anaerobically for 24 h and 48 h. CFU was then measured at each timepoint using the growth conditions described above.

### pH measurements and pH buffered media

pH measurements of bacterial supernatants were performed using a pH meter. Buffered pH medium was made by mixing the medium with filter sterilized buffer K_2_HPO_4_/KH_2_PO_4_ (pH 7.4) to a 22 mM/14 mM final concentration.

### Hydrogen peroxide (H_2_O_2_) measurement

For Amplex Red (Invitrogen Thermo Fisher Scientific, Eugene, OR, USA) assay, all testing reagent solutions were prepared according to manufacturer’s instruction. 1X Reaction Buffer without H_2_O_2_ working solution was used as the negative control and 10 μM H_2_O_2_ was used as the positive control. Diluted test solutions in 1X Reaction Buffer and a volume of 50 µL was used for each reaction. 50 µL of the Amplex Red reagent solution was added to each black/clear bottom microplate well containing the standards, controls, and samples. The plates were incubated at room temperature in the dark for 30 min before the color change was observed. Fluorescence was then measured with a fluorescence microplate reader using excitation at 530 ± 12.5 nm and fluorescence detection at 590 ± 17.5 nm. Background fluorescence, determined for a non-H_2_O_2_ control reaction, is subtracted from each value.

### Effect of H_2_O_2_ on *F. nucleatum* viability

Two concentrations (100 µM and 200 µM) of the H_2_O_2_ were added to the Columbia broth to evaluate their effects on growth of *F. nucleatum* ATCC23726. *F. nucleatum* viability was measured by CFU counting at each timepoint (30 min, 60 min and 120 min). *F. nucleatum* cells on Columbia broth (CB; BD Difco, Detroit, MI, USA) without H_2_O_2_ was used as the control.

### Preparation of *E. faecalis*-incubated PBS solution and catalase rescue assay

To prepare *E. faecalis*-incubated PBS solutions, *E. faecalis* cells were pelleted from an exponential phase culture, washed three times with buffered PBS and resuspended in buffered PBS to an OD600 of 1. The *E. faecalis*-containing PBS solution was then incubated at 37°C under micro-aerobic conditions (N_2_ 90%, CO_2_ 5%, O_2_ 5%) for 2 h and centrifuged (10 min at 17,000 x g, 4°C). The supernatant was collected by filtration (0.22 μm) and used to measure H_2_O_2_ production and *F. nucleatum* killing capacity. For the catalase rescue assay, 2.5 mg of catalase (Thermo Scientific, USA) was added to 1 ml *E. faecalis*-incubated PBS solution or fresh unbuffered PBS solution and incubated at 37°C anaerobically for 12 h. Then, exponential phase *F. nucleatum* cells were added to the solutions, incubated under micro-aerobic conditions for 2 h, and assayed for viability.

### Biofilm based binding and invasion assays

The 35 mm glass bottom dish (Cellvis, CA, USA) was treated with poly-lysine (Electron microscopy sciences, PA, USA) [34]and air dried thoroughly prior to inoculation. To grow *F. nucleatum* biofilm, *F. nucleatum* cells (concentration~ 1 × 10^8^ cells per mL) were seeded into each dish, which contains 1 mL of pre-reduced CB supplemented with hemin at 5 μg per mL and menadione at 1 μg per mL. Each dish was allowed to grow biofilm under anaerobic conditions (5% H_2_, 5% CO_2_, 90% N_2_) at 37°C for 48 h prior before *E. faecalis* cells (concentration ~ 2 × 10^9^ cells per mL) were added for binding and invasion studies. For binding assays, 2 mL of *E. faecalis* (concentration ~ 2 × 10^9^ cells per mL) were added to the *F. nucleatum* biofilm and incubated for 30 min. The unbound *E. faecalis* cells were washed away with pre-reduced CB (three times). The resulting biofilms were scraped off from the dishes, vortexed extensively to be dispersed into single cells, and counted for both *F. nucleatum* and *E. faecalis* respectively within the mixture using the same assays described above. For invasion assays, 2 mL of pre-reduced CB was added to the washed *F. nucleatum* biofilms that have been pre-incubated with *E. faecalis* for 30 min, and incubated anaerobically for another 4 h, 10 h and 24 h before being counted for *F. nucleatum* and *E. faecalis* respectively.

## Results

### *E. faecalis* and *F. nucleatum* binding is mediated by the fusobacterial adhesin Fap2

**Figure 1.**
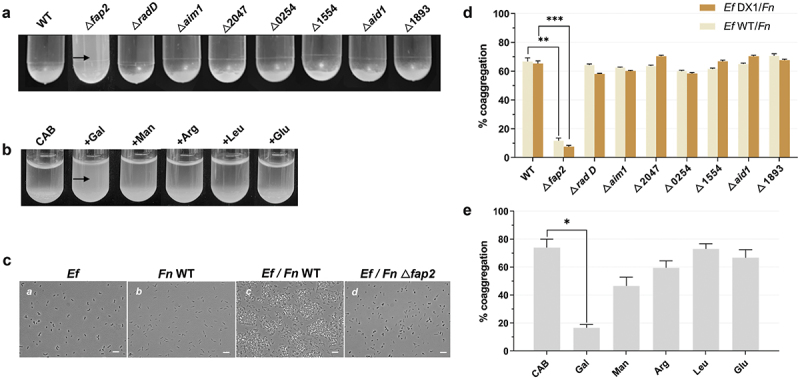
**The qualitative and quantitative coaggregation assay for *E. faecalis* (*Ef*) and *F. nucleatum* (*Fn*)**. (a) Coaggregation assay to screen eight different *F. nucleatum* ATCC23726 outer-membrane protein mutants for defective coaggregation phenotype with *E. faecalis* OG1RF. The arrow points to the *fap2* mutant with binding deficiency. (b) Coaggregation assay to screen sugars and amino acids for their inhibitory efforts on interaction between *E. faecalis* and *F. nucleatum*. The arrow points to galactose with inhibitory effort. (c) Microscopic observation of coaggregation between *E. faecalis* and *F. nucleatum* under different conditions: a. *E. faecalis* alone; b. *F. nucleatum* alone; c. *E. faecalis* and *F. nucleatum*; d. *E. faecalis* and *F. nucleatum fap2*. For each interacting pair, the pictures of 10 random views were taken and 1 representative image was shown. 100X magnification. The scale bar is 10 µm. (d) Spectrophotometric coaggregation assay to quantitatively measure coaggregation between wild type *E. faecalis* (*Ef* WT)/ *E. faecalis* clinical isolate (*Ef* DX1) and wild type *F. nucleatum* (*Fn* WT) and its outer-membrane protein mutants. (e) Spectrophotometric coaggregation assay to quantitatively measure coaggregation between *E. faecalis* and *F. nucleatum* in the presence of selected sugars and amino acids, which are D-galactose (Gal), D-Mannose (Man), L-arginine (Arg), L-leucine (Leu) and L-glutamic acid (Glu). All co-aggregation assays have 120 min incubation time. The data presented are based on experiments conducted in triplicate.

We explored whether *E. faecalis* and *F. nucleatum* could physically bind to each other. Employing a coaggregation assay [28], we showed that *F. nucleatum* wildtype ATCC 23726 coaggregated with *E. faecalis* ATCC type strain OG1RF (Figure 1(a)) under planktonic conditions. Using a quantitative coaggregation assay [32], we further demonstrated that, in addition to *E. faecalis* OG1RF, a clinical isolate, *E. faecalis* DX1, also showed strong coaggregation with *F. nucleatum* ATCC 23726 (Figure 1(d)).

*F. nucleatum* is known to have a set of autotransporter-like outer membrane proteins that interact with other microbes, and our laboratory has previously generated mutants for genes encoding these proteins [28]. To explore the potential mechanism underlying *E. faecalis-F. nucleatum* binding, the entire panel of these outer membrane protein mutants was tested for their coaggregation ability with *E. faecalis*. The qualitative coaggregation measurements shown in Figure 1(a) and Table 1 indicate that only the *fap2* mutant of *F. nucleatum* was defective in binding to *E. faecalis*. Quantitative measurement (Figure 1(d)) confirmed that the *fap2* mutant displayed significantly reduced coaggregation capability with *E. faecalis* compared to wild-type *F. nucleatum*. When observed under the microscope, as shown in Figure 1(c), while *F. nucleatum* and *E. faecalis* monocultures remained suspended as individual cells, large aggregates were present when both species were co-incubated. This coaggregation phenotype was absent when the *F. nucleatum fap2* mutant was co-incubated with *E. faecalis*. These microscopic visual observations further validated the conclusion that *F. nucleatum* binds to *E. faecalis* via its Fap2 adhesin.

**Table 1.** **Scoring coaggregation between *E. faecalis* (*Ef*) with *F. nucleatum* (*Fn*) wildtype (WT) and its mutants**. The scores were determined after 120 min of incubation. Scores were assigned as follows: 0 – no visible co-aggregation; 1 – small aggregates that stay suspended; 2 – larger aggregates that settle slowly and leave the supernatant turbid; 3 – large aggregates that settle quickly but leave the supernatant slightly turbid; 4 – complete sedimentation with a clear supernatant. Experiments were repeated independently at least three times.

	*Fn* WT	Δ*fap2*	Δ*rad D*	Δ*aim1*	Δ2047	Δ0254	Δ1554	Δ*aid1*	Δ1893
Auto-aggregation	0	0	0	0	0	0	0	0	0
*Ef* OG1RF	4	0	4	4	4	4	4	4	4

*F. nucleatum fap2* encodes a galactose-inhibitable adhesin that has been previously shown to be required for hemagglutination, coaggregation, and adherence to mammalian cells [35]. The data above suggest that Fap2 plays a role in mediating the interaction with *E. faecalis* as well. To further confirm this finding, we tested if the observed binding between *E. faecalis* and *F. nucleatum* might be galactose inhibitable. As shown in Figure 1(b) and Table 2, we confirmed that 50 mM galactose indeed resulted in a drastic reduction in the coaggregation between *E. faecalis* and *F. nucleatum*, while other selected carbohydrates or amino acids at the same concentration did not have any significant inhibitory effects. The strong inhibitory effect of galactose on the coaggregation was further supported by a quantitative coaggregation assay (Figure 1(e)).

**Table 2.** **Scoring inhibitory effect of sugars or amino acids on co-aggregation between *E. faecalis and F. nucleatum.*** The scores were determined after 120 min of incubation. Scores were assigned as follows: 0 – no visible coaggregation; 1 – small aggregates that stay suspended; 2 – larger aggregates that settle slowly and leave the supernatant turbid; 3 – large aggregates that settle quickly but leave the supernatant slightly turbid; 4 – complete sedimentation with a clear supernatant. Experiments were repeated independently at least three times.

	CAB	D-Galactose	D-Mannose	L-Arginine	L-Leucine	L-Glutamic acid
*Ef* OG1RF/ *Fn* WT	4	0	3	4	4	4

### *E. faecalis* exerts an inhibitory effect on *F. nucleatum* growth

**Figure 2.**
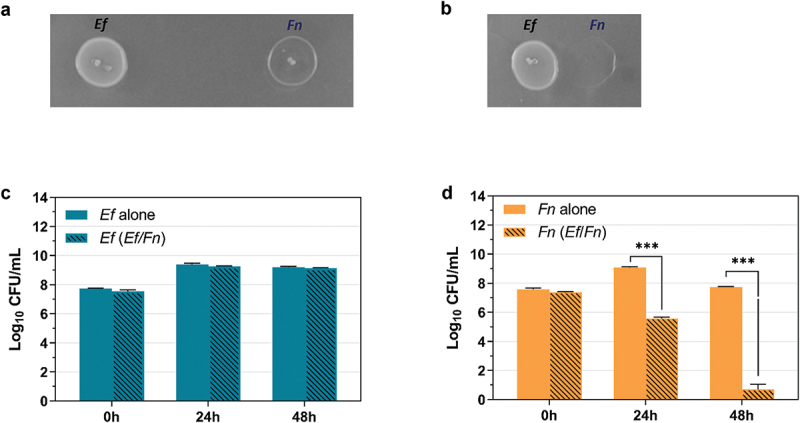
**The qualitative and quantitative analysis of *E. faecalis’* (*Ef*) impact on *F. nucleatum* (*Fn*) growth under different conditions**. (a) Colony competition assay on agar plate to measure the interaction between *E. faecalis* wildtype OG1RF and *F. nucleatum* wildtype ATCC23726 when two bacteria were inoculated more than 10 mm apart. (b) Colony competition assay on plate to measure the interaction between *E. faecalis* and *F. nucleatum* when two bacteria were inoculated right next to each other (less than 1 mm). (c) Competition assay under planktonic condition when *E. faecalis* and *F. nucleatum* were co-incubated for 24 h and 48 h, then CFU of *E. faecalis* in the mixture were counted with selected agar plate in comparison with *E. faecalis* grown in monoculture. (d) Competition assay under planktonic condition when *E. faecalis* and *F. nucleatum* were co-incubated for 24 h and 48 h, then CFU of *F. nucleatum* in the mixture were counted with selected culture in comparison with *F. nucleatum* grown in monoculture. Graph shows means and SD of readings from two individual experiments performed in triplicates. *P < 0.05; ** P < 0.01; *** P < 0.001.

After demonstrating physical binding between *E. faecalis* and *F. nucleatum*, we explored possible physiological interactions between these two bacteria. A plate-based competition assay showed that, when *E. faecalis* and *F. nucleatum* cells were spotted simultaneously in proximity (less than 1 mm apart) on an agar plate, *E. faecalis* exhibited an inhibitory effort against *F. nucleatum* (Figures 2(a,b)). Since there was no overlap of the two bacterial species spotted on the agar surface, the result indicates that the antagonistic effect is mediated through *E. faecalis* production of diffusible molecule(s). Quantitative assays were developed to measure the outcome when *E. faecalis* and *F. nucleatum* were co-aggregated and co-incubated under planktonic conditions. Consistent with the result of plate-based competition assay, growth of *F. nucleatum* in planktonic conditions was significantly inhibited by *E. faecalis* (Figures 2(c,d)). The CFU data shown in Figures 2(c,d) indicate that the inhibitory effort is bactericidal instead of bacteriostatic.

### *E. faecalis* inhibits *F. nucleatum* by generating an acidic environment

**Figure 3.**
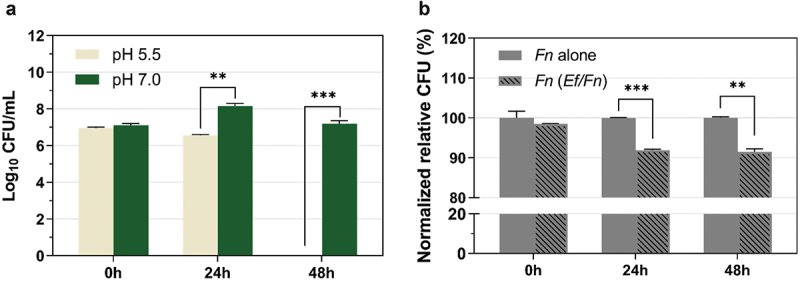
**The acidic environment generated by *E. faecalis* inhibits *F. nucleatum* growth**. (a) pH challenging assay. *F. nucleatum* ATCC 23726 viability was tested under different pH conditions in Columbia broth. The data presented are based on experiments conducted in triplicates. WT, wild type. (b) Competition assay under buffered pH condition. Quantitative analysis of the competition effects between *E. faecalis* (*Ef*) and *F. nucleatum* (*Fn*) under buffered pH condition. Data are expressed as relative CFU (%) compared with the *Fn* alone CFU at each timepoint as 100%. Graph shows means and SD of readings from two individual experiments performed in triplicate. Data represent the means and standard deviation of at least three independent experiments. *P < 0.05; ** P < 0.01; *** P < 0.001. WT, wild type.

To further explore the killing factor(s) of *E. faecalis* against *F. nucleatum*, we tested the pH of monocultures and co-cultures of *E. faecalis* and *F. nucleatum* during exponential growth after starting at pH 7. The pH of *E. faecalis* overnight cultures was around 5.09, the pH of *F. nucleatum* was about 6.39, and the co-culture pH was around 5.33. We examined the growth of *F. nucleatum* at pH 5 and pH 7, and found that the growth of *F. nucleatum* in the pH 5 medium was significantly inhibited when compared to growth in neutral pH medium (Figure 3(a)), suggesting that the low pH environment generated by *E. faecalis* could be one of the inhibitory factors.

To further investigate if low pH may act as an inhibitory factor, we repeated the planktonic competition assay using the same setup described above in a medium buffered by high concentrations of K_2_HPO_4_/ KH_2_PO_4_ to maintain the pH at 7. While *F. nucleatum* survived better in this buffered medium, it still suffered a significant reduction in CFU when co-cultured with *E. faecalis* in comparison with its own monoculture (P < 0.05) (Figure 3(b)). The data indicate that even though the acidic environment generated by *E. faecalis* can play an important role in inhibiting *F. nucleatum* under the conditions tested, there are other killing factor(s) at play.

### *E. faecalis* inhibits *F. nucleatum* by producing H_2_O_2_ under starvation stress conditions

**Figure 4.**
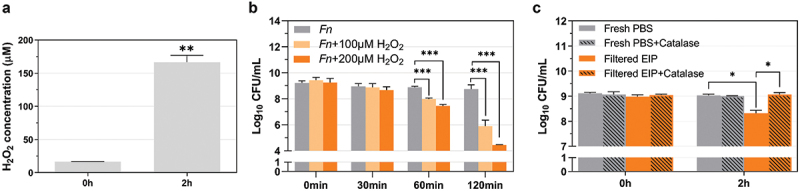
***E. faecalis* (*Ef*) H_2_O_2_ production and its impact on *F. nucleatum* (*Fn*) viability under starvation condition**. (a) AmplexRed assay to measure extracellular H_2_O_2_ production by *E. faecalis* in PBS solution under starvation condition. (b) *F. nucleatum* viability in the presence of H_2_O_2_. Bacterial viability of *F. nucleatum* (CFU) after 30 min, 60 min and 120 min of incubation with 100 µM H_2_O_2_ and 200 µM H_2_O_2_ under starvation condition. (c) *F. nucleatum* viability (CFU) in filtered *E. faecalis-*incubated PBS (EIP) solution or fresh PBS with or without catalase treatment. Graph shows means and SD of readings from two individual experiments performed in triplicate. Columns with the symbol * are statistically different from the control group. *P < 0.05; ** P < 0.01; *** P < 0.001.

It has been well-documented that *E. faecalis* is able to produce H_2_O_2_ [36], which may also contribute to the observed growth inhibition of *F. nucleatum*. When co-cultured with *E. faecalis*, we demonstrated, using the Amplex Red kit, that *E. faecalis* is indeed able to generate H_2_O_2_ (Figure 4(a)). By varying the experimental conditions using different growth media (such as Brain-Heart Infusion, Columbia Broth or PBS) and atomic environments (such as N_2_, CO_2_ or O_2_), we found that *E. faecalis* was able to generate significant amounts of H_2_O_2_ (as high as 167 µM extracellular H_2_O_2_) when incubated in PBS with a brief oxygen exposure or in a micro-aerobic environment (Figure 4(a)).

We then tested *F. nucleatum*’s sensitivity to H_2_O_2_. As shown in Figure 4(b), 100 µM H_2_O_2_ is sufficient to kill *F. nucleatum*, which suggests that H_2_O_2_ generated by *E. faecalis* (167 µM extracellular H_2_O_2_ detected in PBS solution) would be able to kill *F. nucleatum*. For experimental validation, we incubated *E. faecalis* in buffered PBS solution for two hours, collected the supernatant via centrifugation and filtration, then added fresh *F. nucleatum* cells into the filtered supernatants. As expected, Figure 4(c) shows that the *E. faecalis*-incubated PBS solutions exhibited strong killing against *F. nucleatum* in comparison to the fresh PBS solution without *E. faecalis* incubation. Most interestingly, when we treated the *E. faecalis*-incubated PBS solutions with catalase, the resulting solution was no longer able to kill *F. nucleatum* (Figure 4(c)), suggesting H_2_O_2_ is a major killing factor under the tested experimental conditions.

It is worthwhile to mention that pH and H_2_O_2_ based *E. faecalis* killing against *F. nucleatum* under planktonic condition (as described above) seems to be binding independent because *F. nucleatum* wildtype and the *fap2* mutant were found to be killed in the same rate (data not shown). However, the outcome is very different under the biofilm condition as described below.

### *E. faecalis* cells bind and dominate pre-established *F. nucleatum* biofilm

**Figure 5.**
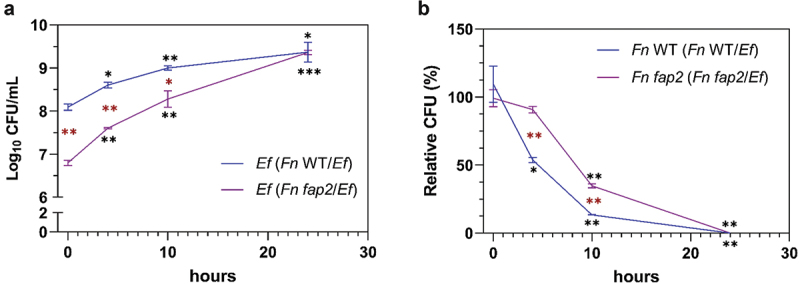
***E. faecalis* (*Ef*) binds and dominates pre-established*F. nucleatum* (*Fn*) biofilms**. (a) The viability of *E. faecalis* wildtype OG1RF (CFU) after binding with the pre-established *F. nucleatum* ATCC23726 biofilms (both wildtype, WT, and the *fap2* mutant) for 30 min then being washed extensively. The resulting biofilms were then incubated in the growth medium for another 4 h, 10 h and 24 h and counted for *E. faecalis* CFU. (b) The viability of *F. nucleatum* after the resulting biofilms were incubated in the growth medium for another 4 h, 10 h and 24 h. Time 0 refers to the timepoint right after the washing step and added fresh medium before the following incubation. Data are expressed as relative percentage CFU via comparing *F. nucleatum* cells from the *E. faecalis* invasion group vs *F. nucleatum* alone as 100% for both WT and the *fap2* mutant, respectively. Graph shows means and SD of readings from experiments performed in duplicates. The black asterisk * denotes statistical difference between the experimental data at 4 h, 10 h and 24 h in comparison with the data at time 0 of the same co-culture (*F. nucleatum* WT*/ E. faecalis or F. nucleatum fap2/ E. faecalis)*. The red asterisk * denotes statistical difference between different cocultures of *F. nucleatum* WT*/ E. faecalis* and *F. nucleatum fap2/ E. faecalis* groups at the same timepoint. *P < 0.05; ** P < 0.01; *** P < 0.001.

The biofilms of *F. nucleatum* wildtype and the *fap2* mutant were pre-established and subjected to the addition of *E. faecalis* wildtype cells. As shown in Figure 5(a), after 30 min incubation followed by extensive washing, a high number of *E. faecalis* cells were still bound to the *F. nucleatum* wildtype biofilm, while significantly lower numbers of *E. faecalis* cells were able to integrate into the preformed *F. nucleatum fap2* biofilm. The data indicate that Fap2-mediated *E. faecalis-F. nucleatum* binding is critical in mediating the integration of *E. faecalis* into the pre-existing *F. nucleatum* biofilm. Meanwhile, when the same *F. nucleatum* biofilms bound with *E. faecalis* were further incubated in growth media for longer periods of time (4, 10 and 24 h), *E. faecalis* exhibited its ability to kill *F. nucleatum* and dominated the biofilm with extensive growth. Compared to wildtype, *F. nucleatum fap2* mutant biofilm cells suffered less viability loss during the first hours (4 and 10 h) of incubation (Figures 5(a,b)), likely due to less bound *E. faecalis* cells to the *fap2* mutant biofilm. However, at 24 h, *E. faecalis* within both *F. nucleatum* wildtype and *fap2* mutant biofilms grew to same biomass, as reflected by the similar CFU, which was accompanied by the almost total loss of viability of both the *F. nucleatum* wildtype and the *fap2* mutant.

## Discussion

There has been extensive research looking at interspecies interactions and investigating their impact on bacterial physiology and disease association within the oral microbiome. However, the studies on the interactions between critical individual microorganisms found at the endodontic infection lesion and their impact on pathogenicity is very limited. Here, we used various *in vitro* assays to demonstrate a strong binding between *E. faecalis* and *F. nucleatum* under both planktonic and biofilm conditions, which is mediated by the *F. nucleatum* outer-membrane protein Fap2. Furthermore, we showed that *E. faecalis* exhibited a strong killing ability against *F. nucleatum* by generating an acidic micro-environment and producing hydrogen peroxide. These interactions may contribute to the observation that *E. faecalis* was able to invade and dominate pre-established *F. nucleatum* biofilms.

Bacteria do not exist as isolated individuals, but rather as members of multispecies communities. To adapt and cope with the ever-changing microenvironment, they need to engage in constant interaction with neighboring residents. Thus, inter-species interactions are the most important processes influencing bacterial physiology, ecology, disease initiation, and disease progression. Our study presents *in vitro* evidence emphasizing the importance of microbial interspecies interaction in the context of endodontic infection.

It has been well documented under clinical conditions that there is often a shift in *E. faecalis* abundance, from being seldom detected to one of the most dominant species within the lesion during the transition from primary to secondary endodontic infection [3,5,8,9,11–13]. Yet it remains to be fully determined how this shift happens. Being able to penetrate dentin tubules, cope with harsh environments such as extensive starvation and resist H_2_O_2_ could contribute to *E. faecalis’s* survival after failed root canal treatments for primary infection and allow *E. faecalis* to gain a competitive advantage in secondary infection [22]. Our study shows that *E. faecalis* displays a strong physical binding to and inhibitory effect against *F. nucleatum*, one of the most dominant pathogens in primary endodontic infection, which may facilitate its colonization and contribute to its increased prevalence in secondary infection.

There are many follow-up questions that need be addressed to obtain a more comprehensive mechanistic understanding of *F. nucleatum-E. faecalis* interaction and its role in endodontic infection progression. This could be partly achieved through genetic and molecular studies to address questions such as: what is the counterpart of *F. nucleatum* Fap2 in *E. faecalis* and how does the physical interaction impact gene expression in *E. faecalis* and *F. nucleatum*.

We fully recognized that the real disease process within the infected root canal is very complex and dynamic, the *in vitro* data presented in this study may not fully recapitulate the *in vivo* situation. Therefore, additional *in vivo* studies are being planned to further validate the hypothesis. We have collected many more clinical samples from the root canals of primary and secondary endodontic infection patients. These samples will be subjected to DNA extraction and deep-sequencing, which will provide additional data for us to further explore the endodontic microbiome shift during the progression of primary to secondary infection. The special attention will be paid to the shift from *F. nucleatum* to *E. faecalis* among these samples. Forsyth has an existing mouse model system where endodontic infection could be induced by injecting endodontic pathogens into mice’s root canals [37]. We plan to use this animal model to conduct *in vivo* studies to explore whether *E. faecalis* is able to attach, invade and dominate a pre-established *F. nucleatum* biofilm within a mouse root canal.

Mouth is the gateway to the GI tract. It is interesting to note that the natural habitat of *E. faecalis* is the gut rather than the oral cavity, yet it manages to colonize inside the tooth root canal and become one of the most dominant endodontic pathogens in secondary infections, through the interaction with *F. nucleatum*. This shows the deep connection between oral and gut microbiome especially under disease conditions. The inhibitory function of *E. faecalis* over *F. nucleatum* may also allow *E. faecalis* to keep *F. nucleatum* in check in the gut.

In conclusion, our data reveal the antagonistic interaction between two key endodontic pathogens, which may help to shed light on microbial shifts from primary to secondary infection. The study also provides future directions to further understand endodontic microbial infections at molecular and genetic levels, which may lead to the development of diagnostic and therapeutic tools against endodontic infections.

## References

[1] Siqueira J, Rocas I. Exploiting molecular methods to explore endodontic infections: part 2—Redefining the endodontic microbiota. J Endod. 2005;31:488–9. 15980706 10.1097/01.don.0000157990.86638.49

[2] Siqueira JF, Rôças IN. Diversity of endodontic microbiota revisited. J Dent Res. 2008;88:969–981. 10.1177/0022034509346549 19828883

[3] Manoil D, Al‐Manei K, Belibasakis GN. A systematic review of the root canal microbiota associated with apical periodontitis: lessons from next‐generation sequencing. Proteomics Clin Appl. 2019;14:1900060. 10.1002/prca.201900060 31950679

[4] Chugal N, Wang J-K, Wang R, et al. Molecular characterization of the microbial flora residing at the apical portion of infected root canals of human teeth. J Endod. 2011;37:1359–1364. 21924182 10.1016/j.joen.2011.06.020 PMC3415298

[5] Siqueira JF, Alves FRF, Rôças IN. Pyrosequencing analysis of the apical root canal microbiota. J Endod. 2011;37:1499–1503. 22000451 10.1016/j.joen.2011.08.012

[6] Özok AR, Persoon IF, Huse SM, et al. Ecology of the microbiome of the infected root canal system: a comparison between apical and coronal root segments. Int Endod J. 2012;45:530–541. 22251411 10.1111/j.1365-2591.2011.02006.x PMC4986919

[7] Persoon IF, Buijs MJ, Özok AR, et al. The mycobiome of root canal infections is correlated to the bacteriome. Clin Oral Invest. 2017;21:1871–1881. 10.1007/s00784-016-1980-3 PMC5442261 27771826

[8] Bouillaguet S, Manoil D, Girard M, et al. Root microbiota in primary and secondary apical periodontitis. Front Microbiol. 2018;9:2374. 30356779 10.3389/fmicb.2018.02374 PMC6189451

[9] Sundqvist G. Associations between microbial species in dental root canal infections. Oral Microbiol Immun. 1992;7:257–262. 10.1111/j.1399-302x.1992.tb00584.x 1494447

[10] Gomes BPFA, Bronzato JD, Almeida-Gomes RF, et al. Identification of *Fusobacterium nucleatum* in primary and secondary endodontic infections and its association with clinical features by using two different methods. Clin Oral Invest. 2021;25:6249–6258. 10.1007/s00784-021-03923-7 33844080

[11] Siqueira JF, Rôças IN. Polymerase chain reaction–based analysis of microorganisms associated with failed endodontic treatment. Oral Surg Oral Med Oral Pathol Oral Radiol Endodontol. 2004;97:85–94. 10.1016/s1079-2104(03)00353-6 14716262

[12] Zandi H, Kristoffersen AK, Ørstavik D, et al. Microbial analysis of endodontic infections in root-filled teeth with apical periodontitis before and after irrigation using pyrosequencing. J Endod. 2018;44:372–378. 29306533 10.1016/j.joen.2017.11.019

[13] Rôças IN, Jung I-Y, Lee C-Y, et al. Polymerase chain reaction identification of microorganisms in previously root-filled teeth in a South Korean population. J Endod. 2004;30:504–508. 15220647

[14] Gomes BPFA, Pinheiro ET, Jacinto RC, et al. Microbial analysis of canals of root-filled teeth with periapical lesions using polymerase chain reaction. J Endod. 2008;34:537–540. 18436030 10.1016/j.joen.2008.01.016

[15] Stuart CH, Schwartz SA, Beeson TJ, et al. *Enterococcus faecalis*: its role in root canal treatment failure and current concepts in retreatment. J Endod. 2006;32:93–98. 16427453 10.1016/j.joen.2005.10.049

[16] Kayaoglu G, Ørstavik D. Virulence factors of *Enterococcus faecalis*: relationship to endodontic disease. Crit Rev Oral Biol M. 2004;15:308–320. 15470268 10.1177/154411130401500506

[17] Khalifa L, Shlezinger M, Beyth S, et al. Phage therapy against *Enterococcus faecalis* in dental root canals. J Oral Microbiol. 2016;8:32157. 27640530 10.3402/jom.v8.32157 PMC5027333

[18] Han YW. *Fusobacterium nucleatum*: a commensal-turned pathogen. Curr Opin Microbiol. 2015;23:141–147. 25576662 10.1016/j.mib.2014.11.013 PMC4323942

[19] Jacinto RC, Montagner F, Signoretti FGC, et al. Microbial interactions, and antimicrobial susceptibility of *Fusobacterium nucleatum* and *Fusobacterium necrophorum* isolated from primary endodontic infections. J Endod. 2008;34:1451–1456. 19026872 10.1016/j.joen.2008.08.036

[20] Pillar CM, Gilmore MS. Enterococcal virulence - pathogenicity island of *E. Faecalis*. Front Biosci. 2004;9:2335. 15353291 10.2741/1400

[21] Gilmore MS, Clewell DB, Ike Y, et al. Enterococci: from commensals to leading causes of drug resistant infection. Boston: Massachusetts Eye and Ear Infirmary; 2014. 24649510

[22] Ran S, Wang J, Jiang W, et al. Assessment of dentinal tubule invasion capacity of *Enterococcus faecalis* under stress conditions ex vivo. Int Endod J. 2015;48:362–372. 24872016 10.1111/iej.12322

[23] Bolstad AI, Jensen HB, Bakken V. Taxonomy, biology, and periodontal aspects of *Fusobacterium nucleatum*. Clin Microbiol Rev. 1996;9:55–71. 8665477 10.1128/cmr.9.1.55 PMC172882

[24] Wu C, Chen Y-W, Scheible M, et al. Genetic and molecular determinants of polymicrobial interactions in *Fusobacterium nucleatum*. Proc Natl Acad Sci. 2021;118:e2006482118. 34074747 10.1073/pnas.2006482118 PMC8201914

[25] Kolenbrander PE, Andersen RN, Moore LV. Coaggregation of *Fusobacterium nucleatum, Selenomonas flueggei, Selenomonas infelix, Selenomonas noxia*, and *Selenomonas sputigena* with strains from 11 genera of oral bacteria. Infect Immun. 1989;57:3194–3203. 2777378 10.1128/iai.57.10.3194-3203.1989 PMC260789

[26] Aral K, Milward MR, Cooper PR. Dysregulation of inflammasomes in human dental pulp cells exposed to *Porphyromonas gingivalis* and *Fusobacterium nucleatum*. J Endod. 2020;46:1265–1272. 32565332 10.1016/j.joen.2020.06.008

[27] Johnson EM, Flannagan SE, Sedgley CM. Coaggregation interactions between oral and endodontic *Enterococcus faecalis* and bacterial species isolated from persistent apical periodontitis. J Endod. 2006;32:946–950. 16982270 10.1016/j.joen.2006.03.023

[28] Kaplan CW, Lux R, Haake SK, et al. The *Fusobacterium nucleatum* outer membrane protein RadD is an arginine‐inhibitable adhesin required for inter‐species adherence and the structured architecture of multispecies biofilm. Mol Microbiol. 2009;71:35–47. 19007407 10.1111/j.1365-2958.2008.06503.x PMC2741168

[29] Brazier JS, Citron DM, Goldstein EJC. A selective medium for *Fusobacterium spp*. J Appl Bacteriol. 1991;71:343–346. 1960109 10.1111/j.1365-2672.1991.tb03798.x

[30] Kolenbrander PE, Andersen RN, Moore LV. Intrageneric coaggregation among strains of human oral bacteria: potential role in primary colonization of the tooth surface. Appl Environ Microb. 1990;56:3890–3894. 10.1128/aem.56.12.3890-3894.1990 PMC185088 2082831

[31] Park J, Shokeen B, Haake SK, et al. Characterization of *Fusobacterium nucleatum* ATCC 23726 adhesins involved in strain-specific attachment to *Porphyromonas gingivalis*. Int J Oral Sci. 2016;8:138–144.

[32] Kaplan A, Kaplan CW, He X, et al. Characterization of aid1, a novel gene involved in *Fusobacterium nucleatum* interspecies interactions. Microbial Ecol. 2014;68:379–387. 10.1007/s00248-014-0400-y PMC4104215 24643713

[33] Kreth J, Merritt J, Shi W, et al. Competition and coexistence between *Streptococcus mutans* and *Streptococcus sanguinis* in the dental biofilm. J Bacteriol. 2005;187:7193–7203. 16237003 10.1128/JB.187.21.7193-7203.2005 PMC1272965

[34] Muchova M, Balacco DL, Grant MM, et al. *Fusobacterium nucleatum* subspecies differ in biofilm forming ability in vitro. Frontiers Oral Heal. 2022;3:853618. 10.3389/froh.2022.853618 PMC8967363 35368312

[35] Coppenhagen-Glazer S, Sol A, Abed J, et al. Fap2 of *Fusobacterium nucleatum* is a galactose-inhibitable adhesin involved in coaggregation, cell adhesion, and preterm birth. Infect Immun. 2015;83:1104–1113. 25561710 10.1128/IAI.02838-14 PMC4333458

[36] Huycke MM, Abrams V, Moore DR. *Enterococcus faecalis* produces extracellular superoxide and hydrogen peroxide that damages colonic epithelial cell DNA. Carcinogenesis. 2002;23:529–536. 11895869 10.1093/carcin/23.3.529

[37] Kawashima N, Niederman R, Hynes R, et al. Infection‐stimulated infraosseus inflammation and bone destruction is increased in P‐/E‐selectin knockout mice. Immunology. 1999;97:117–123. 10447722 10.1046/j.1365-2567.1999.00754.x PMC2326818

